# Factors affecting energy expenditure in a declining fur seal population

**DOI:** 10.1093/conphys/coz103

**Published:** 2019-12-26

**Authors:** Elizabeth A McHuron, Jeremy T Sterling, Daniel P Costa, Michael E Goebel

**Affiliations:** 1 Joint Institute for the Atmosphere and Ocean, University of Washington, 3737 Brooklyn Ave NE, Seattle, WA 98105, USA; 2 Marine Mammal Laboratory, Alaska Fisheries Science Center, National Marine Fisheries Service – NOAA, 7600 Sand Point Way NE, Seattle, WA 98115, USA; 3 Department of Ecology and Evolution, University of California Santa Cruz, 1156 High Street, Santa Cruz, CA 95064, USA

**Keywords:** Doubly labelled water, northern fur seal, otariid, Pribilofs, metabolic rate

## Abstract

Quantifying metabolic rates and the factors that influence them is key to wildlife conservation efforts because anthropogenic activities and habitat alteration can disrupt energy balance, which is critical for reproduction and survival. We investigated the effect of diving behaviour, diet and season on field metabolic rates (FMR) and foraging success of lactating northern fur seals (*Callorhinus ursinus*) from the Pribilof Islands during a period of population decline. Variation in at-sea FMR was in part explained by season and trip duration, with values that ranged from 5.18 to 9.68 W kg^−1^ (*n* = 48). Fur seals experienced a 7.2% increase in at-sea FMR from summer to fall and a 1.9% decrease in at-sea FMR for each additional day spent at sea. There was no effect of foraging effort, dive depth or diet on at-sea FMR. Mass gains increased with trip duration and were greater in the fall compared with summer, but were unrelated to at-sea FMR, diving behaviour and diet. Seasonal increases in at-sea FMR may have been due to costs associated with the annual molt but did not appear to adversely impact the ability of females to gain mass on foraging trips. The overall high metabolic rates in conjunction with the lack of any diet-related effects on at-sea FMR suggests that northern fur seals may have reached a metabolic ceiling early in the population decline. This provides indirect evidence that food limitation may be contributing to the low pup growth rates observed in the Pribilof Islands, as a high metabolic overhead likely results in less available energy for lactation. The limited ability of female fur seals to cope with changes in prey availability through physiological mechanisms is particularly concerning given the recent and unprecedented environmental changes in the Bering Sea that are predicted to have ecosystem-level impacts.

## Teaser

**Lay summary**: We measured metabolic rates of northern fur seals from a declining population and examined the influence of season and foraging behavior on energy expenditure. Our results are relevant for bioenergetic modelling efforts and provide insight into the potential role of prey availability in the population decline.

## Introduction

Wildlife populations currently face a multitude of stressors that can adversely impact population dynamics, including habitat loss, disturbance from human activities and rapidly changing environments. Metabolic rate measurements (or other measures of energy expenditure) are integral in predicting the adverse effects of many of these stressors at both the individual and population levels. For example, they are a key and influential component of bioenergetic models (Winship *et al.*, 2002; Bejarano *et al.*, 2017), which are important in quantifying predator–prey interactions, mitigating human–wildlife conflicts and understanding the influence of prey availability on population dynamics (Wallace *et al.*, 2006; Villegas-Amtmann *et al.*, 2015; Barnett *et al.*, 2017). In addition, anthropogenic disturbance and natural (and human induced) environmental variability often elicit behavioural responses (Wong and Candolin, 2015; Wang *et al.*, 2017), necessitating an understanding of the relationships between energy expenditure and specific behaviours.

Quantifying energy expenditure of free-ranging animals can be difficult, particularly for large carnivores that are wide-ranging and often logistically challenging to capture and handle. The use of doubly labelled water (DLW) remains one of the best techniques for estimating metabolic rates under natural conditions (Nagy *et al.*, 1999; Shaffer, 2011), but it can be infeasible for many species because it typically requires an animal to be captured twice within a period of days to weeks (Speakman, 1997). Metabolic rates of captive animals are thus increasingly used to fill the metabolic data gap and are particularly useful for the ability to isolate the costs of discrete activities and physiological or life history events that can then be applied to estimate the energy requirements of wild populations (Williams *et al.*, 2007; Thometz *et al.*, 2014; Pagano and Williams, 2019). Captive studies cannot, however, mimic the complex behaviour exhibited by free-ranging animals. As a result, they have limited ability to provide insight into how metabolic rates reflect the collective influence of behaviour and life history events.

Adult female otariids are a tractable group for metabolic studies using DLW because their central-place foraging behaviour during lactation facilitates recapture within the measurement interval. Metabolic rates of free-ranging adult females have been quantified in 7 of the 14 extant species, revealing the high cost of existence for all but the two tropical species within this group (Costa and Gentry, 1986; Arnould *et al.*, 1996; Costa and Gales, 2000, 2003; Trillmich and Kooyman, 2001; Villegas-Amtmann *et al.*, 2017; McHuron *et al.*, 2018). Several studies have found that diving behaviour influenced at-sea FMR (Arnould *et al.*, 1996; Costa and Gales, 2000; McHuron *et al.*, 2018), namely dive depth and the percentage of time diving, but others have found no influence of these variables and no differences among individuals using different foraging strategies (Costa and Gales, 2003; Villegas-Amtmann *et al.*, 2017; Jeanniard du Dot *et al.*, 2018; McHuron *et al.*, 2018). Studies on captive animals have detected seasonal changes in metabolic rates, which may be related to intrinsic factors such as molting (Williams *et al.*, 2007; Dalton *et al.*, 2015; Ladds *et al.*, 2017). It is largely unknown, however, whether these same patterns are detectable in free-ranging individuals that exhibit complex behaviours and experience simultaneous physiological stressors.

The goal of this study was to investigate the role of intrinsic and extrinsic factors on at-sea FMR of adult female northern fur seals (*Callorhinus ursinus*). Specifically, we quantified the influence of season, diving behaviour and diet on at-sea FMR using DLW, animal-borne instruments and fatty acid (FA) analysis. We examined how these factors influenced foraging success to better understand the influence of variation in at-sea FMR on the energy available for offspring investment. The eastern stock of northern fur seals has declined by 66% since the 1970s, and while this decline was initially attributed to a female harvest and pelagic sealing, the decline from the 1990s onwards remains unexplained (Towell *et al.*, 2006). Changes in prey availability/distribution leading to reduced pup growth and survival is one hypothesis for the population decline. This hypothesis is primarily based on observations of contrasting pup growth rates (0.9 vs 1.9% day^−1^) and trip durations (5.8–8.9 vs 1.0–3.4 days) between St. Paul Island where the population is declining and Bogoslof Island where the population is increasing (Banks *et al.*, 2006; Kuhn *et al.*, 2014a, 2014b). Thus, in addition to elucidating the factors that affect energy expenditure, there is also a pressing conservation need to quantify metabolic rates and understand the trade-offs between energy expenditure and gain in this population.

**Figure 1:**
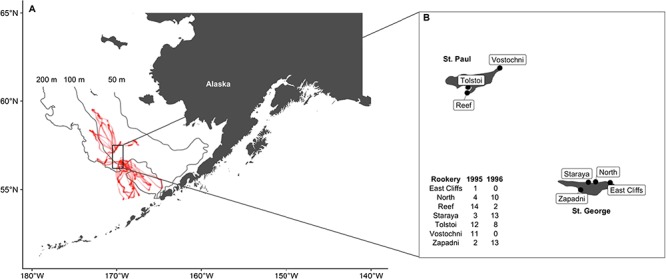
Location of the Pribilof Islands within the Bering Sea, with satellite locations for doubly-labelled water females overlaid on the 200, 100 and 50 m isobaths (**A**), and capture locations of northern fur seals at St. Paul and St. George Islands, with sample sizes by rookery and year (**B**)

## Methods

### Sample collection

**Figure 2:**
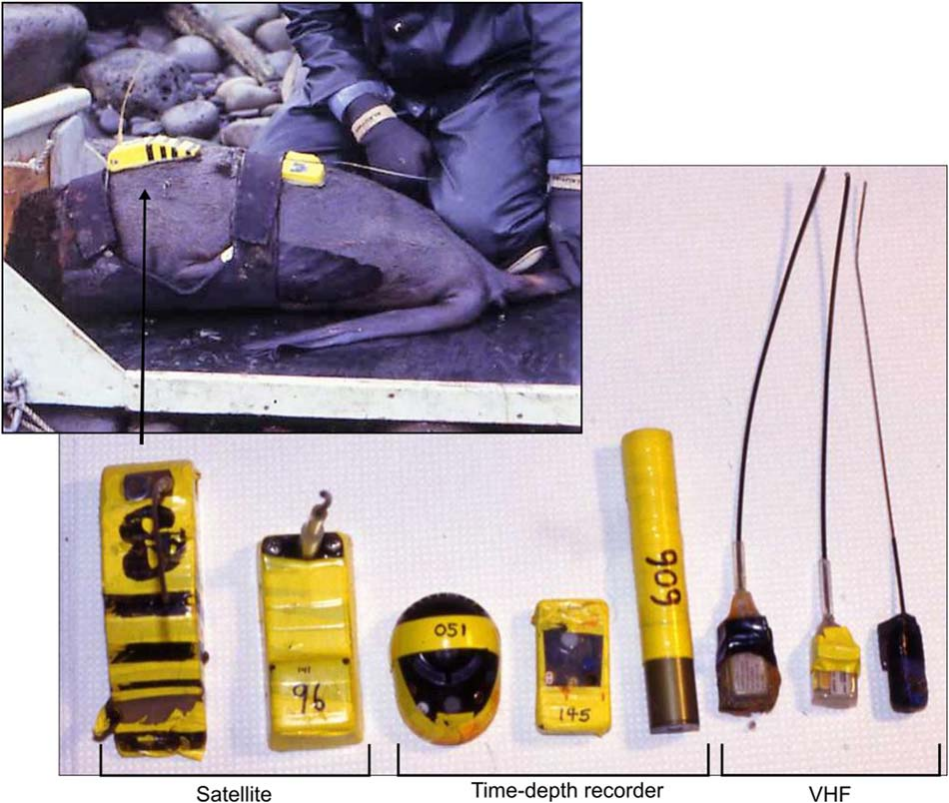
The instruments used in the study and an example of a northern fur seal carrying a satellite tag, time-depth recorder, and VHF tag

Lactating northern fur seals were captured during the summer (July–August) and fall (September–October) of 1995 and 1996 at six rookeries on St. George and St. Paul Islands in Alaska, USA (*n =* 93, Fig. 1, NMFS permit # 837). Once captured in a net, seals were weighed to the nearest 0.1 kg with a Dyna-Link digital scale and physically restrained. Diazepam was administered intramuscularly to facilitate handling and reduce capture stress (0.15 ml, 10 IU ml^−1^). An initial blood sample was collected from an interdigital vein in the hind flipper to determine background isotope concentrations followed by an intraperitoneal injection of a pre-weighed dose of either 10% (55–61 g in 1995, 88–90 g in 1996) or 67% (12–20 g) sterile H_2_^18^O and 3 mL sterile tritiated water containing 1.0 mCi ^3^H. Satellite tags (ST-6, ST-10, Telonics, Mesa, AZ, USA), time-depth recorders (MK-3, MK-5, MK-6, Wildlife Computers, Redmond WA, USA) and VHF tags (ATS, Isanti, MN, USA) were attached to the dorsal pelage mid-back using a quick-setting epoxy (Devcon 5 Minute, Danvers, MA, USA), with each seal receiving variable tag combinations (Table 1, Fig. 2). The time-depth recorders (TDRs) had a resolution of 1 m and were programmed to record depth every 5 or 10 s. Tags were placed in succession along the dorsal midline, with the satellite tag placed first when applicable. Milk samples were collected via manual expression after an intramuscular injection of oxytocin (0.25 mL, 5 IU mL^−1^). Seals were held for approximately 3 h in a ventilated capture box to allow for isotope equilibration (Costa, 1987). A final blood sample was collected before release to determine the equilibration and time zero isotope concentrations. Seals were recaptured after a single foraging trip to sea, with collection of mass, and blood and milk samples as described above. Seals were also given an enema upon recapture unless defecation occurred during handling to obtain hard parts for diet analysis (Supplemental Material). This resulted in diet estimates for a subset of seals, as enemas did not always result in a faecal sample.

**Table 1:** At-sea field metabolic rates (FMR), foraging success and behavioural variables and the tag frontal surface area (FSA) by seal and season

Seal	Season	At-sea FMR (W kg^−1^)	Mass change^a^ (kg)	Water influx (ml kg^−1^ day^−1^)	Trip duration (days)	% dive	Depth (m)	FA cluster	Tag FSA^b^ (cm^2^)
1	Fall	7.79	4.1	184.4	2.8	10.7	45.4	1	7.3
3	Fall	5.18	5.7	160.3	5.2	10.5	45.9	1	12.0
9	Summer	6.48	1.9	202.9	7.0	8.8	84.8	3	16.7
13	Both	6.14, 6.36	−0.2, 12.1	135.9, 140.9	5.8, 9.3	19.0, 12.5	52.1, 34.8	3	17.8
16	Fall	6.89	8.3	197.2	8.7	10.8	12.4	1	12.0
18	Both	8.80, 7.91	4.4, 10.9	138.7, 119.1	5.5, 6.3	14.5, 13.2	34.8, 24.4	3, 2	16.7
22	Fall	6.75	7.4	174.9	7.6	12.7	21.4	3	16.7
25	Summer	6.84	0.5	142.1	6.8	20.8	42.4	3	13.8
35	Fall	7.00	5.0	124.8	2.8	10.7	18.1	3	17.79
60	Fall	7.42	8.8	118.0	8.7	16.4	16.6	3	7.28
61	Fall	6.62	2.4	195.1	8.1	15.3	23.9	2	7.28
67	Fall	7.29	1.0	173.7	9.1	23.9	12.6	1	7.28
70	Fall	6.67	4.9	128.6	7.6	11.3	68.7	2	7.28
74	Fall	6.99	6.4	143.0	9.8	11.1	12.5	3	7.4
77	Fall	6.47	7.2	149.1	6.4	14.2	32.3	2	7.4
343	Both	7.51, 6.90	2.7, 4.6	173.0, 157.2	6.5, 4.8	7.8, 12.8	12.5, 52.6	1, 2	12.0, 7.3
344	Fall	6.46	3.7	183.7	7.2	12.1	11.8	1	12.0
345	Both	6.47, 7.01	3.8, 8.0	168.5, 128.6	7.1, 8.1	9.9, 16.8	7.6, 15.5	1, 3	7.3
349	Both	6.70, 6.90	5.5, 2.9	143.5, 119.9	8.6, 6.9	12.5, 13.8	29.2, 14.4	2, 3	16.5
350	Both	6.20, 7.08	3.7, 6.8	168.9, 201.6	5.7, 7.5	12.1, 18.1	36.2, 11.6	1	16.5
355	Both	7.09, 6.76	5.5, 5.2	181.9, 154.7	6.0, 6.2	9.7, 12.6	32.0, 18.9	2, 3	7.3
356	Summer	6.61	3.9	187.3	7.8	6.9	10.2	1	12.0
357	Fall	6.80	1.7	176.2	5.2	9.2	28.7	2	13.8
360	Both	7.44	3.4, 4.5	196.6, 154.3	3.9, 6.3	15.0, 13.0	38.5, 13.9	2, 3	12.0
361	Both	5.52, 7.61	2.9, 5.9	157.5, 204.8	5.6, 6.5	13.2, 15.4	42.1, 57.9	2	16.45
362	Both	5.50, 7.98	6.9, 4.0	254.1, 225.7	7.9, 7.7	5.7, 8.1	10.5, 8.0	1	7.3
367	Both	5.54, 7.06	6.5, 6.5	212.5, 217.4	6.7, 8.4	16.3, 9.8	11.9, 6.0	1	13.8
370	Both	5.93, 6.48	1.4, 3.7	194.5, 212.0	6.2, 8.0	20.4, 13.4	12.6, 9.2	1	7.3
371	Fall	7.30	5.3	110.8	6.9	15.3	22.3	2	13.8
374	Both	6.77, 8.69	0.0, 7.6	144.0, 141.3	5.0, 6.4	14.8, 10.6	14.5, 15.7	1, 3	7.3
375	Both	6.94, 9.68	4.2, 0.3	153.5, 126.7	6.1, 3.3	15.7, 8.1	17.6, 37.3	1, 2	16.5
376	Both	7.54, 7.55	2.4, 1.7	168.5, 166.6	4.7, 5.8	20.2, 12.8	26.0, 36.1	1, 2	12.0
380	Summer	6.26	5.5	174.0	6.8	16.2	12.8	1	16.5

Summer values are presented first where applicable

a Adjusted mass change as described in text

b The FSA of the anterior tag except where the footprint of the second tag exceeded that of the first. In these cases, the additional FSA was added. For all animals, the FSA of the VHF tag was also added because it was typically offset.

Blood samples were centrifuged to separate the serum component and stored at −20°C until analysis. Milk samples (0.25 mL) were placed in 2 mL of chloroform containing 0.01% butylated hydroxytoluene, and sample tubes were flushed with nitrogen; they were initially frozen at −20°C but were transferred to −80°C at the end of the field season.

### Sample and data analysis

Serum samples were analyzed for ^18^O by Metabolic Solutions (Nashua, NH, USA). The specific activity of ^3^H was determined in duplicate aliquots by scintillation spectrometry as described in Costa *et al.* (1989). Total body water was derived from isotope dilution using ^18^O concentrations and the plateau (initial) and scaling (final) methods. We used the equation from Speakman *et al.* (1993) to calculate CO_2_ production and a value of 23.9 kJ L^−1^ to convert CO_2_ to energy consumption. The resulting estimate of FMR integrates time spent onshore and at-sea. We estimated the at-sea component of FMR using the approach described in Costa and Gales (2003). Water influx was calculated using equations 5 and 6 in Nagy and Costa (1980), which is proxy for prey consumption that is also influenced by prey water content (Costa, 1987).

Satellite locations during a foraging trip were selected between known departure and arrival times determined from VHF telemetry or TDRs. Poor-quality locations (ARGOS ‘B’ and ‘Z’) were removed, and the remaining locations were filtered based on a maximum transit rate of 3 m s^−1^ (R package *argosfilter*, Freitas, 2012). Filtered locations were used to estimate locations at hourly intervals with a time-correlated random walk model (R package *crawl*, Johnson *et al.*, 2008). Dive data were analyzed with Wildlife Computers software (Zero-offset Correction, v1.22, and Dive Analysis, v 4.08). Model fits for the hourly location estimates were used to produce locations for every dive, which were then spatially joined with bathymetric (ETOPO2v2) and Bering Sea shelf water column temperature data (https://www.afsc.noaa.gov/RACE/groundfish/survey_data/ebswater.htm) to classify shelf or pelagic diving and the position of each dive with respect to the mixed layer depth (MLD). We calculated the mean maximum dive depth of dives >3 m, percentage of time spent diving (>3 m) while at sea and the percentage of locations that occurred in pelagic vs continental shelf habitat and above or below the MLD (only seals with satellite tags). Milk samples were analyzed for FA composition as described in Supplemental Material.

While these metrics are relatively simple descriptors of behaviour, we chose them for several key reasons. First, dive depth and the percentage of time spent diving at sea (considered a proxy for foraging effort; Hoskins and Arnould, 2013) have both previously been identified as factors influencing metabolic rates of otariids (Arnould *et al.*, 1996; Costa and Gales, 2000; Hastie *et al.*, 2006; Costa, 2008). These variables also integrate behaviour across the entire foraging trip and thus reflect the same timescale as the energetic measurement. In addition, several of these variables are generally believed to reflect consumption of two common prey species of northern fur seals; dive depth and position within the water column are associated with age-specific consumption of walleye pollock in continental shelf habitat, whereas squid consumption is largely associated with pelagic habitats (Zeppelin and Ream, 2006). While more sophisticated analyses or metrics exist for inferring foraging behaviour from contemporary telemetry and dive tags (Carter *et al.*, 2016), these approaches were largely unavailable to us for a variety of reasons or were inappropriate metrics for this species. For example, only about half of the study animals were wearing satellite tags, precluding the use of state-space models to assign behavioural states to each interpolated location. In addition, we were constrained to using the earliest versions of software available for the data and thus lacked the ability to extract and reanalyze the diving data to compute additional metrics, such as those derived from the entire dive profile (Arthur *et al.*, 2016).

### Statistical analyses

Milk FA clusters were identified using an agglomerative hierarchical cluster analysis of a subset of 19 FAs that represented dietary FAs present in sufficient quantities (≥0.5%; Table S1; Iverson *et al.*, 2004). We used a centred log ratio transformation, the squared Euclidean distance as a measure of similarity, and Ward’s method for clustering (Zeppelin *et al.*, 2015). The number of clusters was identified using a dynamic tree cutting algorithm (Hoskins *et al.*, 2015). We used a linear discriminant analysis to determine which FAs were most important in discriminating among clusters (Fig. S1). Each FA sample was then associated with the scat/enema sample and behavioural data representative of that foraging trip to understand how FA clusters reflected foraging behaviour (Fig. S1, Supplemental Material). To increase sample size, we used all milk samples collected in 1995 and 1996 (*n* = 291 trips). Similarly, clusters were described using diving (*n* = 191 trips) and tracking data (*n* = 99 trips) and scat/enema samples (*n =* 79 trips) from all seals instrumented in the study years.

Linear mixed effects models were used to examine the effect of season and foraging behaviour on at-sea FMR, with individual included as a random effect to account for repeated samples within the same year. We did not include year or island in the model because these two variables were confounded due to sample design and most useable measurements occurred in 1996 (see Results). Instrumentation has been shown to affect diving metabolic rates in captive northern fur seals (Rosen *et al.*, 2018), presumably due to increases in hydrodynamic drag. To account for any potential effects of varying instrument size (Fig. 2), we included the frontal surface area (FSA) of the tag(s) as a covariate. While FSA may not be the best representation of the drag an animal experiences (Kay *et al.*, 2019; Kyte *et al.*, 2019), it has been used previously for this species (Skinner *et al.*, 2012) and was the best metric we could reliably calculate given the dataset and the free-ranging nature of seals in our study. We calculated the total FSA (sum across all tags) and the effective FSA (the FSA of the anterior tag and any excess area by the second tag) to account for the fact that TDRs were placed directly behind the often larger satellite tag. We only present results using the effective FSA because the overall patterns were the same (Table 1). Foraging behaviour variables included in the model were mean maximum dive depth, the percentage of time spent diving and the FA cluster of the milk sample collected at the time of recapture. The inclusion of FA cluster likely incorporated the variability associated with island or year, as spatial and temporal variability in diet are common within this population (Antonelis *et al.*, 1997; Zeppelin and Ream, 2006). We used model averaging of all candidate models with a cumulative sum weight of 95% to assess the effect of each variable on at-sea FMR. Variables were considered important when the 95% CI did not include zero. Model assumptions were assessed using residual and quantile plots of the full model.

We used a similar approach to determine the factors influencing foraging success, which we quantified using three variables: absolute mass gain, daily mass gain at sea, and water influx. We did not use a mixed effects approach for the two mass gain variables because the variance of the random effect in all models was estimated at exactly zero. Because seals spent variable amounts of time onshore, we adjusted mass to better reflect mass gain at sea using data on the fasting mass loss ashore during summer and fall from another fur seal species (Georges and Guinet, 2000). Explanatory variables included in the full model for each metric of foraging success included season, initial mass, trip duration, tag FSA, at-sea FMR and FA cluster. As above, we used model averaging to assess the effect of each variable on foraging success. All analyses were conducted using R v 3.4.1 (R Core Team 2017, https://www.R-project.org/). Means are presented ± SD.

## Results

**Figure 3:**
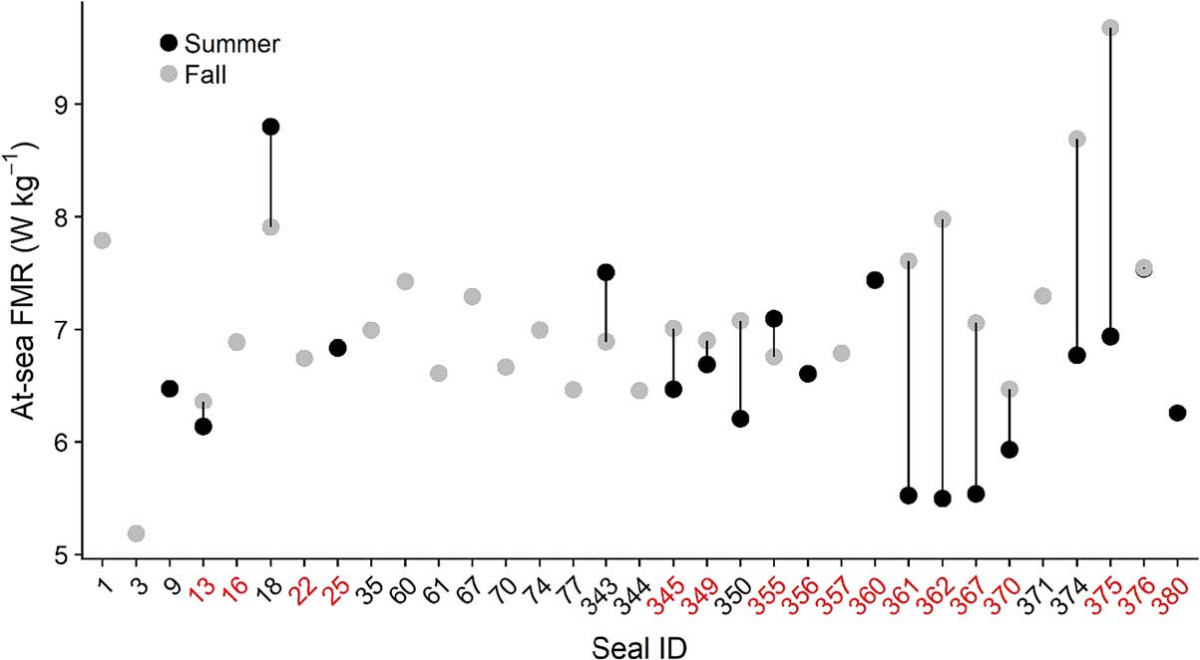
At-sea field metabolic rates (W kg^−1^) of 33 northern fur seals. Seal ID is colour-coded based on whether seals were sampled in a single season (black) or during both summer and fall (red)

Metabolic rates were obtained for all fur seals but a subset of measurements (*n* = 45) were excluded because isotope levels at recapture were too close to background to yield reliable estimates of energy expenditure. The final dataset consisted of 48 measurements from 33 seals that ranged in body mass from 26.0 to 47.6 kg (Tables 1, S2, Fig. 3). The average absolute and daily adjusted mass gains were 4.6 ± 2.7 kg (−0.2–12.1 kg) and 0.7 ± 0.4 kg day^−1^ (−0.03–1.8 kg day^−1^), respectively. Seals foraged in continental shelf and pelagic habitats up to 350 km from the colony (Fig. 1A). They spent an average of 13.2 ± 3.8% (5.7–23.9%) of their time diving to depths >3 m, with the vast majority of diving activity occurring at night (81.8 ± 12.2%). Seals dove to average maximum depths of 26.2 ± 17.4 m, with means for individual seals ranging from 6.0–84.8 m (Table 1).

Foraging trips were assigned to one of four FA clusters, although none of the samples collected from DLW seals were classified into Cluster 4 (Table 1, Fig. S1). Squid appeared to be the predominate prey species of seals with trips in Cluster 1, whereas variation in the proportion of age-zero pollock vs mature pollock appeared to distinguish seals in Cluster 2 and 3, respectively (Supplemental Material).

**Figure 4:**
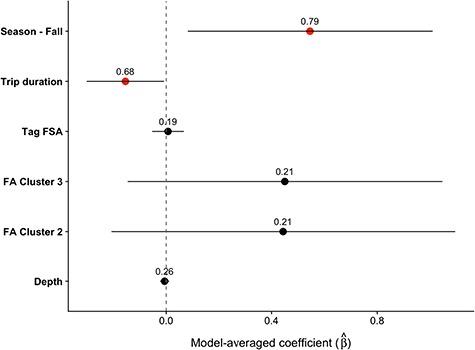
Model averaged conditional coefficients with 95% CI for at-sea field metabolic rate (W kg^−1^). Points are shown in red when the CI did not include zero. Numbers above each point represent the sum of weight for each variable. The intercept estimate was 7.36 (5.94–8.78)

**Figure 5:**
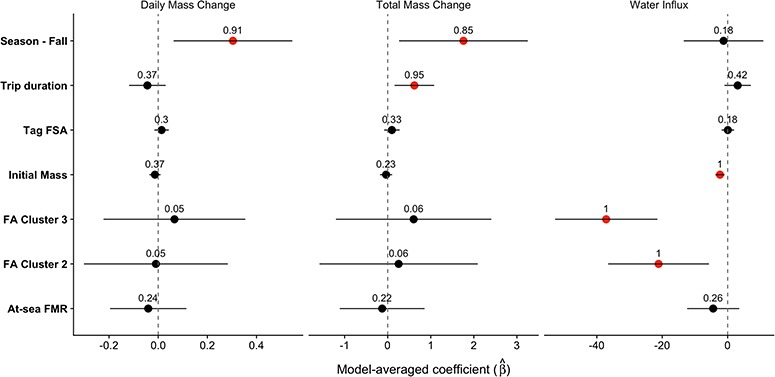
Model averaged conditional coefficients with 95% CI for the daily rate of mass change (kg), total mass change across the foraging trip (kg) and water influx (ml kg^−1^ day^−1^). Points are shown in red when the CI did not include zero. Numbers above each point represent the sum of weight for each variable. Intercept estimates (95% CI) are as follows: daily mass change—0.86 (−0.23–1.95), total mass change—−0.05 (−6.17–6.08), water influx—271.5 (195.5–347.6)

Individual models for at-sea FMR explained between 1.0–26% of the variability in the data (Fig. 4, Table S3). Fur seals experienced a 7.2% increase in at-sea FMR from summer to fall and a 1.9% decrease in at-sea FMR for each additional day spent at sea (Fig. 4). There was no effect of foraging behaviour or tag FSA on at-sea FMR. Total mass gain increased with trip duration and season, with seals gaining an additional 0.6 kg per additional foraging day and an average of 1.8 kg more during the fall. The daily rate of mass gain increased an average of 0.3 kg day^−1^ between summer and fall (Fig. 5, Tables S4–S5). Water influx was not influenced by trip duration, but it varied among FA clusters. The highest estimates of water influx were for seals in FA Cluster 1, followed by FA Cluster 2 and lastly by FA Cluster 3 (Fig. 5, Table S6), which is consistent with relative water content estimates of putative prey. Tag FSA was not important in any of the foraging metric models.

## Discussion

Metabolic rates collected from free-ranging animals provide insight into the collective influence of animal behaviour and physiology on energy expenditure. The life history and behaviour of female otariids lend themselves to metabolic studies, allowing us to quantify the influence of diving behaviour, diet and season on a species that has experienced an unexplained population decline since the 1990s. While data for this study were collected over two decades ago, our comparatively large sample size has nearly doubled the available metabolic data for this species. As such, our dataset contributes to our knowledge of the energetics of free-ranging mammals and is directly relevant to the conservation of northern fur seals.

Individual variation in time-activity budgets is an important driver of intraspecific variation in FMR since foraging is an energetically expensive activity (Gorman *et al.*, 1998; Acevedo-Gutiérrez *et al.*, 2002; Goldbogen *et al.*, 2011; Williams *et al.*, 2014). Despite this, we did not find a relationship between at-sea FMR and the relative time seals spent diving, a proxy for foraging effort. This is perhaps not surprising given that a relationship between at-sea FMR and relative time spent diving has been detected for Antarctic fur seals and New Zealand sea lions (but in opposite directions) and is apparently absent for other otariids (Arnould *et al.*, 1996; Costa and Gales, 2000, 2003; Villegas-Amtmann *et al.*, 2017; McHuron *et al.*, 2018). These contrasting relationships may be due to differences in the underlying mechanisms driving variation in the percentage of time spent diving, and how time at sea is allocated to other behaviours. Northern fur seals feed predominately at night; thus, a significant portion of their foraging trip is allocated towards other activities such as grooming, resting and transiting (Battaile *et al.*, 2015). Considerable advancements in tagging technology have occurred since our study, which could be used to address the limitations of our study and better characterize the time-activity budgets during foraging trips. There have been several more recent studies on energy expenditure of northern fur seals that coupled accelerometers with metabolic measurements (Jeanniard-du-Dot *et al.*, 2017; Jeanniard du Dot *et al.*, 2018), but they were largely not focused on examining the factors influencing the rate of energy expenditure.

Seasonal changes in metabolic rates associated with the timing of molt have been measured in both terrestrial and marine species, with increases attributed to the added energetic cost of tissue generation and thermoregulation (Dietz *et al.*, 1992; Boily and Lavigne, 1997; Hoye and Buttemer, 2011; Dalton *et al.*, 2015; Ladds *et al.*, 2017). In northern fur seals, captive juveniles experienced a 50% (resting metabolic rate) and 16% (daily energy expenditure) increase during the fall compared with other seasons, which the authors hypothesized was due to the direct costs of molting (Dalton *et al.*, 2015). The molt of adult female northern fur seals lasts approximately 15 weeks, with an average mid-date of molt of October 18–November 13 depending on age (Scheffer and Johnson, 1963). This timing corresponds to our sample collection dates, suggesting that at-sea metabolic rates may have been higher in the fall compared with summer because females were molting. The timing of this increase also coincides with seasonal increases in lactation costs (Donohue *et al.*, 2002) that can result in increased foraging effort (Hoskins and Arnould, 2013), but this seems an unlikely explanation given we did not find any relationships between at-sea FMR and diving behaviour or foraging success.

Central-place foragers can compensate for higher energy demands by increasing foraging effort, trip duration or switching to energy rich prey, none of which are mutually exclusive (Georges and Guinet, 2000; Shaffer *et al*., 2003; Beauplet *et al*., 2004; McDonald *et al*., 2009; Hoskins and Arnould, 2013). Increases in trip duration across lactation appear to be a shared trait among many fur seal species, including northern fur seals (Boyd *et al*., 1991; Gentry, 1998; Georges and Guinet, 2000; Goldsworthy, 2006); the average trip durations of seals in our study increased by a maximum of 0.3 and 0.85 days between summer and fall in 1995 and 1996, respectively. The decrease in at-sea FMR associated with longer trips is likely associated with changes in time-activity budgets (Arnould *et al*., 1996; Jeanniard-du-Dot *et al*., 2017); trip durations typically increase with travel distance from the colony, and thus, females may spend a greater percentage of time in transit or other behaviours on longer foraging trips. Seasonal changes in at-sea FMR did not affect any of the foraging success metrics, but the positive relationship between total mass gain and trip duration suggests the benefits associated with increasing trip duration appear to outweigh the metabolic costs associated with additional time spent at sea. The ability to increase trip duration to cope with higher energy demands is, however, not limitless, as females must balance trade-offs among trip duration, the fasting ability of the pup and the potential decline in milk energy delivery rate relative to time spent at sea (Costa, 2008).

The time- and cost-intensive nature of metabolic studies using free-ranging animals often results in the energetic requirements of a species being quantified from a few studies, which is problematic if there is spatial or temporal variation in factors affecting metabolic rates. Dietary variation at multiple temporal and spatial scales is present within this population (Antonelis *et al.*, 1997; Zeppelin and Ream, 2006), and variable consumption of age-zero vs adult pollock has previously been attributed to inter-annual variation in at-sea FMR of northern fur seals (Costa and Gentry, 1986; Costa, 2008). We found no evidence that FA cluster or dive depth had any influence on at-sea FMR, despite that FA clusters appeared to capture inter-island and temporal dietary variation related to variable consumption of squid, and age-zero and mature pollock. While the conclusions of Costa (2008) were based on a relatively small number of animals with unknown foraging behaviour, they are consistent with findings by Costa and Gales (2000) and Hastie *et al.* (2006) that increases in dive depth were associated with reduced metabolic rates. Our study was conducted during the initial period of unknown population decline, and it may be that females had reached their metabolic ceiling, thus minimizing any diet-related effects, as has been suggested for Antarctic fur seals (Costa, 2008). The metabolic rates reported here were similar to a 2011 study that found no effect of foraging strategies on at-sea FMR (Jeanniard du Dot *et al.*, 2018), providing corroborating evidence that this population may currently have limited energetic flexibility to respond to future environmental changes. In a conceptual model of parental attendance, Costa (2008) suggested that female otariids should first increase foraging effort (at-sea FMR) before increasing trip duration in response to changes in food availability because of the negative impact of longer trip durations on pup growth rates. Further investigation of the links between female foraging behaviour, pup growth and prey availability and the impacts on population dynamics is thus warranted, particularly given the contrasting pup growth rates and trip durations between islands experiencing divergent population trends.

### Conclusion

The eastern Pacific stock of northern fur seals has experienced an unexplained population decline since the 1990s, with pup production estimates in 2018 at their lowest in over 100 years (Towell *et al.*, 2019). There is thus a pressing need to understand the factor/s contributing to the decline, and in particular, the role that food limitation might play given that one of their primary prey resources during lactation is also the target species for the US largest fishery. Metabolic rate measurements are critical to the conservation of this species because they provide insight into how hard seals are working to find food and how energy needs might change in a temporally and spatially dynamic environment. They are also a key parameter in bioenergetic models that can be used to quantify population-level prey consumption, which is needed to better understand prey needs and potential overlap with fishing activities. In this study, we were able to nearly double the available metabolic data for this species, with data collection occurring during the initial population decline. We detected seasonal increases in at-sea FMR that coincided with the timing of the annual molt, but foraging behaviour appeared to have little impact on energy expenditure of northern fur seals. A large portion of the variation in at-sea FMR remained unexplained, which could be due to a combination of introduced variability associated with the method itself, other factors not considered here, or our inability to partition time-activity budgets into discrete behaviours. Future studies are thus warranted, particularly as the Bering Sea has experienced unprecedented warming with winter ice extent at record lows in the last 2 years that will likely have ecosystem-level impacts (Stabeno and Bell, 2019). Despite study limitations, our data indicate that female northern fur seals appear to have reached a metabolic ceiling early in the current population decline, which may be a contributing factor to the lower pup growth rates observed in the Pribilof Islands compared with Bogoslof Island where fur seals have experienced healthy population growth. These high metabolic rates required a female to spend more of her energy gain on her own metabolic overhead, with no indication that individuals with higher metabolic rates were able to gain more mass while at sea without increasing their trip durations. While they do not provide direct support for the hypothesis that food limitation is contributing to the population decline, the results of our study provide indirect evidence that food limitation is likely reducing the amount of energy available a female has to invest in lactation with adverse consequences for reproductive success.

## Supplementary Material

Energy_Expenditure_Supplemental_Material_R1_coz103

## References

[ref1] Acevedo-Gutiérrez A, Croll DA, Tershy BR (2002) High feeding costs limit dive time in the largest whales. J Exp Biol 205: 1747–1753. 12042333 10.1242/jeb.205.12.1747

[ref2] Antonelis GA, Sinclair EH, Ream RR, Robson BW (1997) Inter-island variation in the diet of female northern fur seals (*Callorhinus ursinus*) in the Bering Sea. J Zool 242: 435–451.

[ref3] Arnould J, Boyd IL, Speakman JR (1996) The relationship between foraging behavior and energy expenditure in Antarctic fur seals. J Zool 239: 769–782.

[ref4] Arthur B, Hindell M, Bester MN, Oosthuizen WC, Wege M, Lea MA (2016) South for the winter? Within-dive foraging effort reveals the trade-offs between divergent foraging strategies in a free-ranging predator. Funct Ecol 30: 1623–1637.

[ref5] Banks A, Iverson S, Springer A, Ream R, Sterling J (2006) Consequences of fur seal foraging strategies (COFFS). North Pacific Resarch Board Final Report 414.

[ref6] Barnett A, Braccini M, Dudgeon CL, Payne NL, Abrantes KG, Sheaves M (2017) The utility of bioenergetics modelling in quantifying predation rates of marine apex predators: ecological and fisheries implications. Sci Rep 1–10. 29021551 10.1038/s41598-017-13388-y PMC5636836

[ref7] Battaile BC, Sakamoto KQ, Nordstrom CA, Rosen DAS, Trites AW (2015) Accelerometers identify new behaviors and show little difference in the activity budgets of lactating northern fur seals (*Callorhinus ursinus*) between breeding islands and foraging habitats in the eastern Bering Sea. PLoS One 10: 1–20. 10.1371/journal.pone.0118761 PMC4373933 25807552

[ref8] Beauplet G, Dubroca L, Guinet C, Cherel Y, Dabin W, Gagne C, Hindell M (2004) Foraging ecology of subantarctic fur seals *Arctocephalus tropicalis* breeding on Amsterdam Island: seasonal changes in relation to maternal characteristics and pup growth. Mar Ecol Prog Ser 273: 211–225.

[ref9] Bejarano AC, Wells RS, Costa DP (2017) Development of a bioenergetic model for estimating energy requirements and prey biomass consumption of the bottlenose dolphin *Tursiops truncatus*. Ecol Modell 356: 162–172.

[ref10] Boily P, Lavigne DM (1997) Developmental and seasonal changes in resting metabolic rates of captive female grey seals. Can J Zool 75: 1781–1789.

[ref11] Boyd I, Lunn N, Barton T (1991) Time budgets and foraging characteristics of lactating Antarctic fur seals. J Anim Ecol 60: 577–592.

[ref12] Carter MID, Bennett KA, Embling CB, Hosegood PJ, Russell DJF (2016) Navigating uncertain waters: a critical review of inferring foraging behaviour from location and dive data in pinnipeds. Mov Ecol 4: 1–20. 27800161 10.1186/s40462-016-0090-9 PMC5080796

[ref13] Costa D (1987) Isotopic methods for quantifying material and energy intake of free-ranging marine mammals In Costa D, Worthy G, Castellini M, eds, Approaches to Marine Mammal Energetics. Society for Marine Mammalogy, Lawrence, KS, pp. 43–66

[ref14] Costa DP (2008) A conceptual model of the variation in parental attendance in response to environmental fluctuation: foraging energetics of lactating sea lions and fur seals. Aquat Conserv Mar Freshw Ecosyst 17: S44–S52.

[ref15] Costa DP, Croxall JP, Duck CD (1989) Foraging energetics of Antarctic fur seals in relation to changes in prey availability. Ecology 70: 596–606.

[ref16] Costa DP, Gales NJ (2000) Foraging energetics and diving behavior of lactating New Zealand sea lions. *Phocarctos hookeri*. J Exp Biol 203: 3655–3665. 11060226 10.1242/jeb.203.23.3655

[ref17] Costa DP, Gales NJ (2003) Energetics of a benthic diver: seasonal foraging ecology of the Australian sea lion, *Neophoca cinerea*. Ecol Monogr 73: 27–43.

[ref18] Costa DP, Gentry RL (1986) Reproductive energetics of northern fur seals In Gentry RL, Kooyman GL, eds, Fur Seals: Maternal Strategies on Land and at Sea. Princeton University Press, Princeton, NJ, USA, pp. 79–101

[ref19] Dalton AJM, Rosen DAS, Trites AW (2015) Resting metabolic rate and activity: key components of seasonal variation in daily energy expenditure for the northern fur seal (*Callorhinus ursinus*). Can J Zool 93: 635–644.

[ref20] Dietz MW, Daan S, Masman D (1992) Energy requirements for molt in the kestrel *Falco tinnunculus*. Physiol Zool 65: 1217–1235.

[ref21] Donohue MJ, Costa DP, Goebel E, Antonelis GA, Baker JD (2002) Milk intake and energy expenditure of free-ranging northern fur seal, *Callorhinus ursinus*, pups. Physiol Biochem Zool 75: 3–18. 11880973 10.1086/338284

[ref22] Freitas C (2012) argosfilter: Argos locations filter. R package version 0.63. https://CRAN.R-project.org/package=argosfilter.

[ref23] Gentry RL (1998) Behavior and Ecology of the Northern Fur Seal. Princeton University Press, Princeton, NJ, USA

[ref24] Georges JY, Guinet C (2000) Maternal care in the subantarctic fur seals on Amsterdam Island. Ecology 81: 295–308.

[ref25] Goldbogen JA, Calambokidis J, Oleson E, Potvin J, Pyenson ND, Schorr G, Shadwick RE (2011) Mechanics, hydrodynamics and energetics of blue whale lunge feeding: efficiency dependence on krill density. J Exp Biol 214: 131–146. 21147977 10.1242/jeb.048157

[ref26] Goldsworthy SD (2006) Maternal strategies of the New Zealand fur seal: evidence for interannual variability in provisioning and pup growth strategies. Aust J Zool 54: 31–44.

[ref27] Gorman ML, Mills MG, Raath JP, Speakman JR (1998) High hunting costs make African wild dogs vulnerable to kleptoparasitism by hyaenas. Nature 852: 1992–1994.

[ref28] Hastie GD, Rosen DAS, Trites AW (2006) The influence of depth on a breath-hold diver: predicting the diving metabolism of Steller sea lions (*Eumetopias jubatus*). J Exp Mar Bio Ecol 336: 163–170.

[ref29] Hoskins AJ, Arnould JPY (2013) Temporal allocation of foraging effort in female Australian fur seals (*Arctocephalus pusillus doriferus*). PLoS One 8: e79484. 24244511 10.1371/journal.pone.0079484 PMC3828376

[ref30] Hoskins AJ, Costa DP, Wheatley KE, Gibbens JR, Arnould JPY (2015) Influence of intrinsic variation on foraging behaviour of adult female Australian fur seals. Mar Ecol Prog Ser 526: 227–239.

[ref31] Hoye BJ, Buttemer WA (2011) Inexplicable inefficiency of avian molt ? Insights from an opportunistically breeding arid-zone species, *Lichenostomus penicillatus*. PLoS One 6, e16230. doi: 10.1371/journal.pone.0016230. PMC3032729 21311594

[ref32] Iverson S, Field C, Bowen W, Blanchard W (2004) Quantitative fatty acid signature analysis: a new method of estimating predator diets. Ecol Monogr 74: 211–235.

[ref33] Jeanniard-du-Dot T, Trites AW, Arnould JPY, Speakman JR, Guinet C (2017) Activity-specific metabolic rates for diving, transiting, and resting at sea can be estimated from time – activity budgets in free-ranging marine mammals. Ecol Evol 7: 2969–2976. 28479996 10.1002/ece3.2546 PMC5415512

[ref34] Jeanniard du Dot T, Trites AW, Arnould JPY, Speakman JR, Guinet C (2018) Trade-offs between foraging efficiency and pup feeding rate of lactating northern fur seals in a declining population. Mar Ecol Prog Ser 600: 207–222.

[ref35] Johnson D, London J, Lea M, Durban J (2008) Continuous-time correlated random walk model for animal telemetry data. Ecology 89: 1208–1215. 18543615 10.1890/07-1032.1

[ref36] Kay WP, Naumann DS, Bowen HJ, Withers SJ, Evans BJ, Wilson RP, Stringell TB, Bull JC, Hopkins PW, Börger L (2019) Minimizing the impact of biologging devices: using computational fluid dynamics for optimizing tag design and positioning. Methods Ecol Evol 2019: 1222–1233.

[ref37] Kuhn CE, Baker JD, Towell RG, Ream RR (2014a) Evidence of localized resource depletion following a natural colonization event by a large marine predator. J Anim Ecol 83: 1169–1177. 24450364 10.1111/1365-2656.12202

[ref38] Kuhn CE, Ream RR, Sterling JT, Thomason JR, Towell RG (2014b) Spatial segregation and the influence of habitat on the foraging behavior of northern fur seals (*Callorhinus ursinus*). Can J Zool 92: 861–873.

[ref39] Kyte A, Pass C, Pemberton R, Sharman M, McKnight JC (2019) A computational fluid dynamics (CFD) based method for assessing the hydrodynamic impact of animal borne data loggers on host marine mammals. Mar Mammal Sci 35: 364–394.

[ref40] Ladds MA, Slip DJ, Harcourt RG (2017) Intrinsic and extrinsic influences on standard metabolic rates of three species of Australian otariid. Conserv Physiol 5: 1–14. 10.1093/conphys/cow074 PMC5570045 28852504

[ref41] McDonald BI, Goebel ME, Crocker DE, Tremblay Y, Costa DP (2009) Effects of maternal traits and individual behavior on the foraging strategies and provisioning rates of an income breeder, the Antarctic fur seal. Mar Ecol Prog Ser 394: 277–288.

[ref42] McHuron EA, Peterson SH, Hückstädt LA, Melin SR, Harris JD, Costa DP (2018) The energetic consequences of behavioral variation in a marine carnivore. Ecol Evol 8: 4340–4351. 29721302 10.1002/ece3.3983 PMC5916299

[ref43] Nagy KA, Costa DP (1980) Water flux in animals: analysis of potential errors in the tritiated water method. Am J Physiol 238: R454–R465. 6990797 10.1152/ajpregu.1980.238.5.R454

[ref44] Nagy KA, Girard IA, Brown TK (1999) Energetics of free-ranging mammals, reptiles, and birds. Annu Rev Nutr 19: 247–277. 10448524 10.1146/annurev.nutr.19.1.247

[ref45] Pagano AM, Williams TM (2019) Estimating the energy expenditure of free-ranging polar bears using tri-axial accelerometers: a validation with doubly labeled water. Ecol Evolece3.5053. 10.1002/ece3.5053 PMC6468055 31015999

[ref46] Rosen DAS, Gerlinsky CG, Trites AW (2018) Telemetry tags increase the costs of swimming in northern fur seals, *Callorhinus ursinus*. Mar Mammal Sci 34: 385–402.

[ref47] Scheffer VB, Johnson AM (1963) Molt in the northern fur seal. United States Fish and Wildlife Servies Special Scientific Report. 1–34.

[ref48] Shaffer SA (2011) A review of seabird energetics using the doubly labeled water method. Comp Biochem Physiol Part A 158: 315–322. 10.1016/j.cbpa.2010.07.012 20656049

[ref49] Shaffer SA, Costa DP, Weimerskirch H (2003) Foraging effort in relation to the constraints of reproduction in free-ranging albatrosses. Funct Ecol 17: 66–74.

[ref50] Skinner JP, Burkanov VN, Andrews RD (2012) Influence of environment, morphology, and instrument size on lactating northern fur seal *Callorhinus ursinus* foraging behavior on the Lovushki Islands, Russia. Mar Ecol Prog Ser 471: 293–308.

[ref51] Speakman JR (1997) Doubly Labelled Water: Theory and Practice. Chapman & Hall, London, UK

[ref52] Speakman JR, Nair KS, Goran MI (1993) Revised equations for calculating CO_2_ production from doubly labeled water in humans. Am J Physiol 264: E912–E917. 8333517 10.1152/ajpendo.1993.264.6.E912

[ref53] Stabeno PJ, Bell SW (2019) Extreme conditions in the Bering Sea (2017–2018): record-breaking low sea-ice extent. Geophys Res Lett 46: 8952–8956.

[ref54] Thometz NM, Tinker MT, Staedler MM, Mayer KA, Williams TM (2014) Energetic demands of immature sea otters from birth to weaning: implications for maternal costs, reproductive behavior and population-level trends. J Exp Biol 217: 2053–2061. 24920834 10.1242/jeb.099739

[ref55] Towell R, Ream R, Sterling J, Bengston J, Williams M (2019) 2018 Northern Fur Seal Pup Production and Adult Male Counts on the Pribilof Islands, Alaska. Memorandum for the Record. Alaska Fisheries Science Center, mml: 1–5. https://www.fisheries.noaa.gov/resource/data/2018-northern-fur-seal-pup-production-and-adult-male-counts-pribilof-islands-alaska.

[ref56] Towell RG, Ream RR, York AE (2006) Decline in northern fur seal (*Callorhinus ursinus*) pup production on the Pribilof Islands. Mar Mammal Sci 22: 486–491.

[ref57] Trillmich F, Kooyman GL (2001) Field metabolic rate of lactating female Galapagos fur seals (*Arctocephalus galapagoensis*): the influence of offspring age and environment. Comp Biochem Physiol Part A 129: 741–749. 10.1016/s1095-6433(01)00343-9 11440861

[ref58] Villegas-Amtmann S, McDonald BI, Páez-Rosas D, Aurioles-Gamboa D, Costa DP (2017) Adapted to change: low energy requirements in a low and unpredictable productivity environment, the case of the Galapagos sea lion. Deep Res Part II 140: 94–104.

[ref59] Villegas-Amtmann S, Schwarz LK, Sumich JLL, Costa DP (2015) A bioenergetics model to evaluate demographic consequences of disturbance in marine mammals applied to gray whales. Ecosphere 6: 1–19.

[ref60] Wallace BP, Kilham SS, Paladino FV, Spotila JR (2006) Energy budget calculations indicate resource limitation in Eastern Pacific leatherback turtles. Mar Ecol Prog Ser 318: 263–270.

[ref61] Wang Y, Smith JA, Wilmers CC (2017) Residential development alters behavior, movement, and energetics in an apex predator, the puma. PLoS One 12: e0184687. 29020087 10.1371/journal.pone.0184687 PMC5636101

[ref62] Williams TM, Rutishauser M, Long B, Fink T, Gafney J, Mostman-Liwanag H, Casper D (2007) Seasonal variability in otariid energetics: implications for the effects of predators on localized prey resources. Physiol Biochem Zool 80: 433–443. 17508338 10.1086/518346

[ref63] Williams TM, Wolfe L, Davis T, Kendall T, Richter B, Wang Y, Bryce C, Elkaim GH, Wilmers CC (2014) Instantaneous energetics of cougar kills reveals advantage of felid sneak attacks. Science (80-) 346: 81. 10.1126/science.1254885 25278610

[ref64] Winship A, Trites A, Rosen D (2002) A bioenergetic model for estimating the food requirements of Steller sea lions *Eumetopias jubatus* in Alaska, USA. Mar Ecol Prog Ser 229: 291–312.

[ref65] Wong BBM, Candolin U (2015) Behavioral responses to changing environments. Behav Ecol 26: 665–673.

[ref66] Zeppelin TK, Johnson DS, Kuhn CE, Iverson SJ, Ream RR (2015) Stable isotope models predict foraging habitat of northern fur seals (*Callorhinus ursinus*) in Alaska. PLoS One 10: 1–21. 10.1371/journal.pone.0127615 PMC4451762 26030280

[ref67] Zeppelin TK, Ream RR (2006) Foraging habitats based on the diet of female northern fur seals (*Callorhinus ursinus*) on the Pribilof Islands, Alaska. J Zool 270: 565–576.

