# Supplementary material for: Factors affecting energy expenditure in a declining fur seal population
Source: Conserv Physiol. 2019 Dec 26;7(1):coz103. doi: 10.1093/conphys/coz103 (PMC6933311; doi:10.1093/conphys/coz103)
Supplement: Energy_Expenditure_Supplemental_Material_R1_coz103 [file energy_expenditure_supplemental_material_r1_coz103.docx]

**Supplemental Material: Factors affecting energy expenditure in a declining fur seal population**

Elizabeth A. McHuron, Jeremy T. Sterling, Daniel P. Costa, and Michael E. Goebel

*Fatty acid analysis*

Lipids were extracted using the Folch method (Folch *et al.*, 1957), filtered, and dried over anhydrous sodium sulfate. Fatty acid methyl esters (FAME) were prepared from ≥ 100 mg of pure extracted lipid using 1.5 mL 8% boron trifluoride in methanol (v/v) and 1.5 mL of hexane, capped in nitrogen, and heated at 100 ºC for one hour. FAME were extracted into hexane, concentrated under nitrogen, and brought up to volume in high purity hexane (50 mg ml^-1^). FAME were analyzed in duplicate using temperature-programmed capillary gas-liquid chromatography on a Perkin Elmer Autosystem II Capillary FID fitted with a 30 m x 0.25 mm column coated with 50% cyanopropyl polysiloxane (0.25 μm film, J & W, DB-23, Folsom, CA). The abundance of each fatty acid (FA) was calculated as the percentage by weight of the total extractable triacylglycerol fatty acids. Fatty acids are designated by carbon chain length:number of double bonds and the position (n-x) of the first double bond with respect to the methyl end.

*Enema analysis*

Enemas were given to females upon recapture by administering approximately 1 L of warmed water into the colon through a flexible plastic tube. Fecal material was collected into clear plastic garbage bags when the water was naturally expelled. As described in Zeppelin and Ream (2006), fecal samples were rinsed through a series of nested sieves and identifying prey features (fish bones, fish otoliths, and cephalopod beaks) were identified to the lowest possible taxon using a reference collection. The relative abundance of each species was calculated based on the frequency at which it occurred in each sample (FO). The FO of each prey type by FA cluster was calculated by dividing the number of enema samples that contained that prey species by the total number of samples for each cluster. We did not quantify the relative contribution of different age-classes of pollock in enema samples because scat and enema samples are biased towards smaller age classes, whereas older pollock are more likely to occur in spew samples (Gudmundson *et al.*, 2000).

*Description of FA Clusters*

As described in the main text, milk FA samples were analyzed using a hierarchical cluster analysis to identify FA clusters. FA clusters were qualitatively described using diving behavior, enema samples, and the prevalence of each strategy by island and year to understand how clusters related to diet. With respect to diving behavior, we calculated the mean maximum dive depth, percentage of time spent diving in continental shelf and pelagic habitats, and the percentage of dives below the mixed layer depth (continental shelf dives only) for each foraging trip. Each trip was associated with the FA sample (and resulting cluster) that represented that foraging trip, and diving variables were averaged across all trips that occurred within each FA cluster. Four FA clusters were identified, but because none of the DLW females were classified into Cluster 4, we limit our discussion to the first three clusters. Milk samples were classified to the correct cluster with 89% accuracy, with c18:4n-1, c18:1n-5, c18:2n-6, and c21:5n-3 contributing the most to discrimination among clusters along the first two axes (Fig. S1A).

The prey species that occurred most frequently in enema samples were walleye pollock (*Theragra chalcogramma*) and squid spp., consisting mainly of *Gonatus* spp. and *Berryteuthis magister*. Squid occurred frequently in samples from females classified to Cluster 1 but were infrequent in the diet of seals classified to Clusters 2 or 3 (Fig. S1B). Pollock was present in every sample collected from Cluster 2 females, 89% of Cluster 3 females, and 53% of Cluster 1 females. Other species were found infrequently in enema samples, including Atka mackerel (*Pleurogrammus monopterygius*), capelin (*Mallotus villosus*), Pacific cod (*Gadus macrocephalus*), greenlings (Hexagrammidae), Pacific herring (*Clupea pallasi*), sablefish (*Anoplopoma fimbria*), Pacific sand lance (*Ammodytes hexapterus*), and northern smoothtongue (*Leuroglossus schmidti*).

Milk samples classified to Cluster 1 were predominately associated with shallow foraging in pelagic habitats, whereas Clusters 2 and 3 were associated with continental shelf habitats (Fig. S1C). Diving depths of seals in Cluster 3 tended to be greater than those of Cluster 2 (Fig. S1C), with a greater proportion of dives below the mixed layer depth (24% vs <10% for both years). The frequency of the three clusters varied among years and islands, with Clusters 1 and 3 prevalent in 1995 (and Cluster 2 in 1996), and Cluster 1 more prevalent at St. George compared with St. Paul in both years (Fig. S1D).

Temporal and spatial variation in diet has been well documented for northern fur seals (Antonelis *et al.*, 1997; Zeppelin and Ream, 2006; Zeppelin *et al.*, 2015), which is primarily driven by differences in habitat availability and environmental characteristics that affect the abundance and distribution of prey species. St. George Island is closer to the edge of the continental shelf than many of the rookeries on St. Paul Island, thus, species that are common in oceanic habitats, such as squid, tend to be more important in the diet of St. George compared with St. Paul seals (Antonelis *et al.*, 1997; Zeppelin and Ream, 2006). The abundance and distribution of juvenile walleye pollock is in part driven by the extent of the cold pool and mixing dynamics (Mueter *et al.*, 2006; Coyle *et al.*, 2011). The diving behavior of fur seals is largely reflective of the species and age-composition of their primary prey. For example, age-zero pollock primarily occupy continental shelf habitat within the mixed layer depth (MLD), whereas more mature pollock are found below the MLD or on the bottom and diving behavior reflects the vertical distribution of the dominant pollock age class (Sterling, 2009). The MLD in 1995 was shallow and well-stratified and the cold pool extended well onto the shelf. In contrast, 1996 was characterized by a less stratified and deeper MLD and the extent of the cold pool was much smaller (Fig. S2). The abundance of age-zero pollock was higher in 1996 compared with 1995 (Brodeur *et al.*, 2002), which is consistent with the abundance of age-zero pollock in scat and spew samples collected from 1996 (MML unpublished data). In contrast, older pollock occurred more frequently in the diet of fur seals in 1995 compared with 1996 (MML unpublished data).

Collectively, these data suggest that milk samples classified to Cluster 1 were associated with a diet comprised of squid, juvenile pollock, and to a lesser extent other species primarily found in oceanic habitats. Cluster 2 appeared primarily associated with foraging on juvenile pollock, whereas females in Cluster 3 likely targeted more mature pollock in addition to younger age classes and other fish species. These conclusions also align with the water flux analysis that indicated FA cluster significantly influenced water influx (Fig. 4); squid have an average water content that ranges from ~82 – 88% compared with ~78 – 84% for pollock, with lower water content in adult compared with younger pollock (Perez, 1994; Van Pelt *et al.*, 1997; Sinclair *et al.*, 2015).

**Literature Cited**

Antonelis GA, Sinclair EH, Ream RR, Robson BW (1997) Inter-island variation in the diet of female northern fur seals (*Callorhinus ursinus*) in the Bearing Sea. *J Zool London* 242: 435–451.

Brodeur RD, Wilson MT, Ciannelli L, Doyle M, Napp JM (2002) Interannual and regional variability in distribution and ecology of juvenile pollock and their prey in frontal structures of the Bering Sea. *Deep Res Part II Top Stud Oceanogr* 49: 6051–6067.

Coyle KO, Eisner LB, Mueter FJ, Pinchuk AI, Janout MA, Cieciel KD, Farley E V., Andrews AG (2011) Climate change in the southeastern Bering Sea: Impacts on pollock stocks and implications for the oscillating control hypothesis. *Fish Oceanogr* 20: 139–156.

Folch J, Lees M, Stanley GHS (1957) A simple method for the isolation and purification of total lipids from animal tissues. *J Biol Chem* 226: 497–509.

Gudmundson CJ, Zeppelin TK, Ream RR (2000) Application of two methods for determining diet of northern fur seals (*Callorhinus ursinus*). *Fish Bull* 104: 445–455.

Mueter FJ, Ladd C, Palmer MC, Norcross BL (2006) Bottom-up and top-down controls of walleye pollock (*Theragra chalcogramma*) on the Eastern Bering Sea shelf. *Prog Oceanogr* 68: 152–183.

Perez MA (1994) Calorimetry measurements of energy value of some Alaskan fishes and squids. *NOAA Tech Memo* NMFS-AFSC-: 32.

Sinclair EH, Walker WA, Thomason JR (2015) Body size regression formulae, proximate composition and energy density of eastern Bering Sea mesopelagic fish and squid. *PLoS One* 11: 1–13.

Sterling JT (2009) Northern Fur Seal Foraging Behaviors, Food Webs, and Interactions with Oceanographic Features in the Eastern Bering Sea. University of Washington. PhD Dissertation.

Van Pelt TI, Piatt JF, Lance BK, Roby DD (1997) Proximate composition and energy density of some north pacific forage fishes. *Comp Biochem Physiol Part A Physiol* 118: 1393–1398.

Zeppelin TK, Johnson DS, Kuhn CE, Iverson SJ, Ream RR (2015) Stable isotope models predict foraging habitat of northern fur seals (*Callorhinus ursinus*) in Alaska. *PLoS One* 10: 1–21.

Zeppelin TK, Ream RR (2006) Foraging habitats based on the diet of female northern fur seals (*Callorhinus ursinus*) on the Pribilof Islands, Alaska. *J Zool* 270: 565–576.


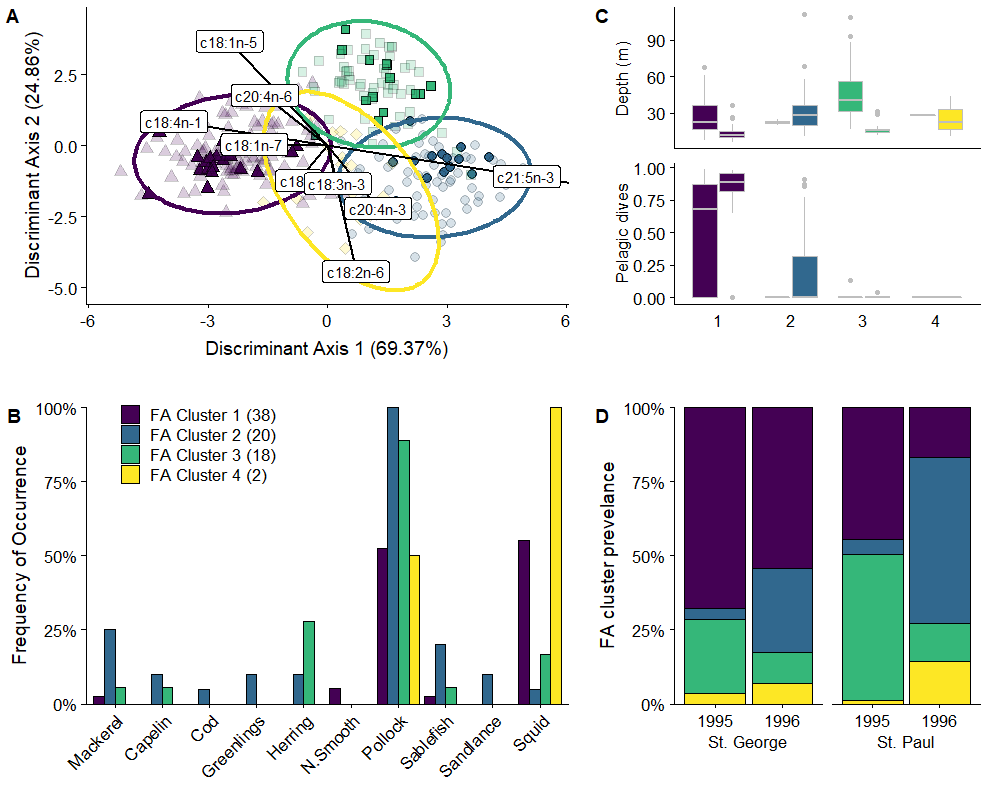


Figure S1. Linear discriminant analysis showing which fatty acids (FA) were most important in discriminating among milk FA samples (A), and differences among clusters in % Frequency of Occurrence of prey species obtained from enema samples (B), dive depth and the proportion of pelagic dives (C), and prevalence of clusters between years and islands (D)

*
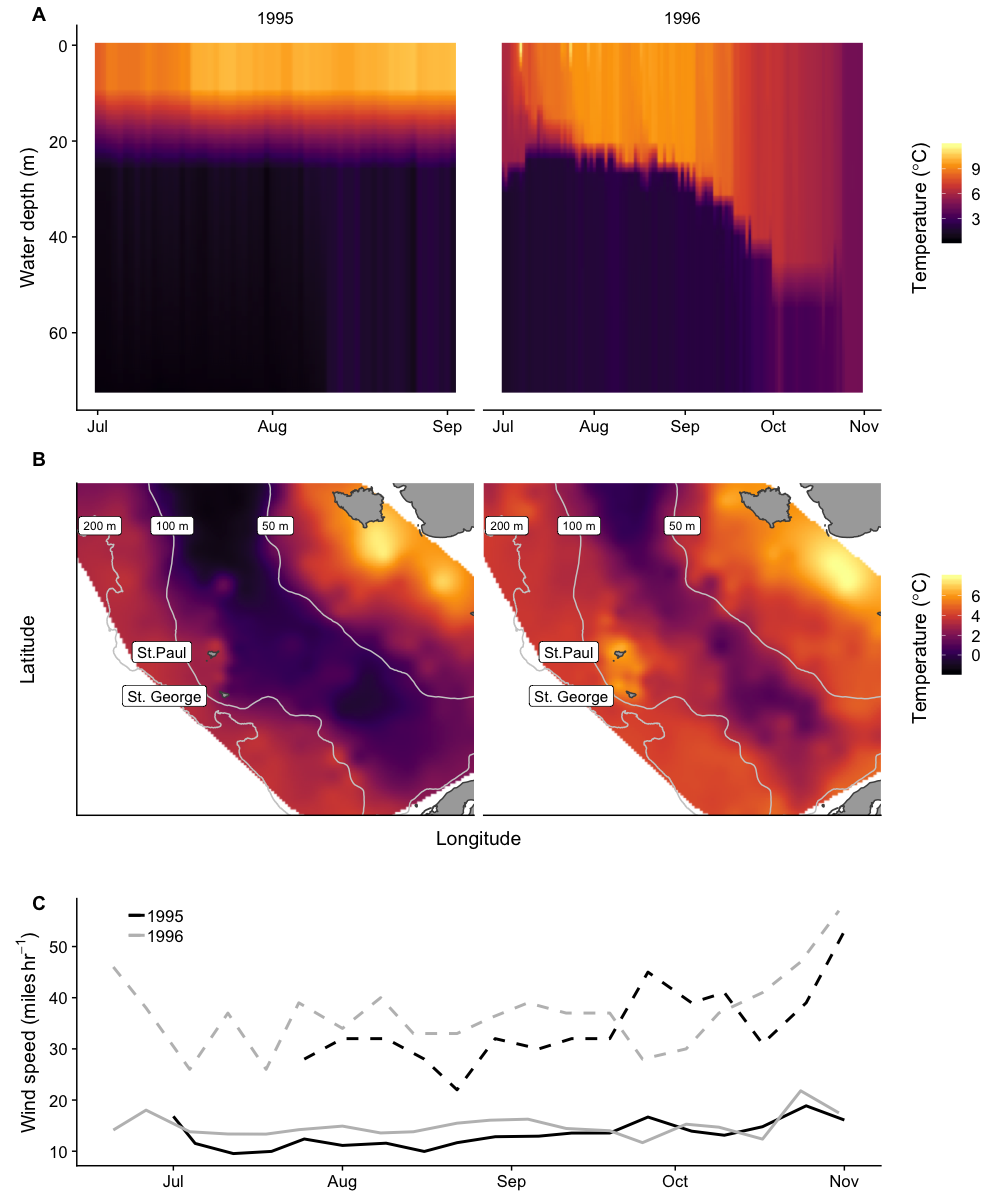
*

Figure S2. Plots of environmental conditions in the Bering Sea during 1995 and 1996. A) Water temperatures between July and November from the M4 buoy (https://www.ndbc.noaa.gov). B) Bottom water temperatures obtained from groundfish surveys conducted between June and July in each year showing the extent of the cold pool (< 2 ºC, https://www.afsc.noaa.gov/RACE/groundfish/survey_data/ebswater.htm). C) Average daily (solid) and maximum wind speeds (dashed) recorded at the weather station on St. Paul Island (www.ncdc.noaa.gov).

Table S1. The 19 dietary fatty acids used to identify clusters from milk samples collected from lactating northern fur seals.

| Fatty acid |
| --- |
| c14.0 |
| c16.0 |
| c16.1n7 |
| c18.0 |
| c18.1n5 |
| c18.1n7 |
| c18.1n9 |
| c18.2n6 |
| c18.3n3 |
| c18.4n1 |
| c18.4n3 |
| c20.1n9 |
| c20.4n3 |
| c20.4n6 |
| c20.5n3 |
| c21.5n3 |
| c22.1n11 |
| c22.1n9 |
| c22.6n3 |

Table S2. Measurement interval, mass, proportion of measurement interval at sea, water influx, carbon dioxide production, and energy expenditure measurements of northern fur seals (*n* = 33). The island (St. Paul – SP, St. George – SG), year (1995, 1996), and season (Summer – S, Fall – F, Both - B) of the measurements are also shown. Summer values are presented first when applicable.

| Seal ID | Location/Time | Interval  (days) | Initial Mass  (kg) | Final Mass  (kg) | Prop Sea | Water Influx  (ml kg^-1^ day^-1^) | CO_2_  (ml g^-1^ hr^-1^) | FMR  (W kg^-1^) | At-sea FMR  (W kg^-1^) |
| --- | --- | --- | --- | --- | --- | --- | --- | --- | --- |
| 1 | SP – 95 - F | 4.9 | 27.5 | 28.5 | 0.58 | 184.4 | 0.98 | 6.51 | 7.79 |
| 3 | SP – 95 - F | 7.1 | 46.5 | 47.5 | 0.74 | 160.3 | 0.66 | 4.39 | 5.18 |
| 9 | SP – 95 - S | 8.2 | 33.0 | 33.0 | 0.86 | 202.9 | 0.91 | 6.05 | 6.48 |
| 13 | SP – 95 - B | 7.6,11.8 | 45.0,40.5 | 41.0, 47.0 | 0.76, 0.79 | 135.9, 140.9 | 0.81, 0.86 | 5.40, 5.72 | 6.14, 6.36 |
| 16 | SP – 95 - F | 8.9 | 26.0 | 34.0 | 0.98 | 197.2 | 1.03 | 6.83 | 6.89 |
| 18 | SP – 95 – B | 7.8, 8.6 | 34.5, 33.0 | 35.0, 39.5 | 0.70, 0.73 | 138.7, 119.1 | 1.19,1.07 | 7.89, 7.09 | 8.80, 7.91 |
| 22 | SP – 95 – F | 10.0 | 32.5 | 35.5 | 0.76 | 174.9 | 0.91 | 6.02 | 6.75 |
| 25 | SP – 95 - S | 8.0 | 36.5 | 35.0 | 0.85 | 142.1 | 0.96 | 6.38 | 6.84 |
| 35 | SP – 95 - F | 4.0 | 35.5 | 38.0 | 0.7 | 124.8 | 0.92 | 6.08 | 7.00 |
| 60 | SP – 96 - F | 10.1 | 43.5 | 49.0 | 0.87 | 118.0 | 1.06 | 7.03 | 7.42 |
| 61 | SP – 96 - F | 8.8 | 35.0 | 36.0 | 0.92 | 195.1 | 0.96 | 6.37 | 6.62 |
| 67 | SP – 96 - F | 9.8 | 36.5 | 36.0 | 0.92 | 173.7 | 1.06 | 7.05 | 7.29 |
| 70 | SP – 96 - F | 9.8 | 45.5 | 45.0 | 0.78 | 128.6 | 0.90 | 6.00 | 6.67 |
| 74 | SP – 96 - F | 11.8 | 36.5 | 39.0 | 0.83 | 143.0 | 0.98 | 6.48 | 6.99 |
| 77 | SP – 96 - F | 8.9 | 42.0 | 43.5 | 0.72 | 149.1 | 0.85 | 5.62 | 6.47 |
| 343 | SG – 96 - B | 8.9, 6.7 | 39.6, 36.0 | 37.8, 36.8 | 0.74, 0.71 | 173.0, 157.2 | 1.01, 0.91 | 6.71, 6.01 | 7.51, 6.90 |
| 344 | SG – 96 - F | 7.8 | 46.0 | 48.0 | 0.92 | 183.7 | 0.94 | 6.22 | 6.46 |
| 345 | SG – 96 - B | 8.8 | 45.0, 42.4 | 43, 48.6 | 0.72, 0.92 | 168.5, 128.6 | 0.85, 1.02 | 5.62, 6.77 | 6.47, 7.01 |
| 349 | SG – 96 - B | 8.9, 8.9 | 36.4, 42.0 | 41.4, 40.4 | 0.97, 0.78 | 143.5, 119.9 | 0.99, 0.94 | 6.60, 6.23 | 6.70, 6.90 |
| 350 | SG – 96 - B | 6.9, 8.9 | 44.6, 47.6 | 45.6, 50.8 | 0.82, 0.85 | 168.9, 201.6 | 0.85, 1.00 | 5.66, 6.62 | 6.20, 7.08 |
| 355 | SG – 96 - B | 7.2, 7.7 | 35, 36 | 38.4, 38.2 | 0.83, 0.81 | 181.9, 154.7 | 0.99, 0.93 | 6.57, 6.18 | 7.09, 6.76 |
| 356 | SG – 96 - S | 8.9 | 34.0 | 36.0 | 0.87 | 187.3 | 0.94 | 6.22 | 6.61 |
| 357 | SG – 96 - F | 5.9 | 37.2 | 37.6 | 0.89 | 176.2 | 0.97 | 6.46 | 6.80 |
| 360 | SG – 96 - B | 4.0, 7.7 | 35.6, 35.0 | 38.8, 36.6 | 0.98, 0.81 | 196.6, 154.3 | 1.11, 1.03 | 7.38, 6.85 | 7.44 |
| 361 | SG – 96 - B | 6.2, 6.8 | 44.6, 42.4 | 46.0, 47.4 | 0.89, 0.95 | 157.5, 204.8 | 0.78, 1.12 | 5.19, 7.45 | 5.52, 7.61 |
| 362 | SG – 96 - B | 8.2, 8.6 | 34, 34.2 | 40.4, 36.4 | 0.97, 0.90 | 254.1, 225.7 | 0.81, 1.16 | 5.41, 7.67 | 5.50, 7.98 |
| 367 | SG – 96 - B | 8.6, 8.9 | 34.0, 34.4 | 37.2, 39.8 | 0.77, 0.94 | 212.5, 217.4 | 0.73, 1.04 | 4.84, 6.88 | 5.54, 7.06 |
| 370 | SG – 96 - B | 7.1, 8.9 | 34.2, 33.4 | 34.0, 35.4 | 0.87, 0.90 | 194.5, 212.0 | 0.83, 0.93 | 5.53, 6.17 | 5.93, 6.48 |
| 371 | SG – 96 - F | 9.8 | 45.8 | 44.0 | 0.71 | 110.8 | 0.97 | 6.41 | 7.30 |
| 374 | SG – 96 - B | 6.7, 7.0 | 39.2, 37.4 | 43.6, 36.0 | 0.74, 0.91 | 144.0, 141.3 | 0.90, 1.27 | 5.98, 8.41 | 6.77, 8.69 |
| 375 | SG – 96 - B | 8.0, 3.7 | 40.6, 42 | 41.2, 41.4 | 0.77, 0.89 | 153.5, 126.7 | 0.94, 1.41 | 6.24, 9.34 | 6.94, 9.68 |
| 376 | SG – 96 - B | 5.9, 6.7 | 33.0, 34.6 | 33.4, 34.6 | 0.87, 0.89 | 168.5, 166.6 | 1.08, 1.09 | 7.14, 7.21 | 7.54, 7.55 |
| 380 | SG – 96 - S | 8.6 | 40.2 | 42.0 | 0.78 | 174.0 | 0.84 | 5.59 | 6.26 |

Table S3. Summary of model parameters for all models included in model averaging for at-sea FMR of lactating northern fur seals in 1995 and 1996, including marginal (*r^2^_m_*) and conditional (*r^2^_c_*) estimates.

| *Model* | *(Intercept)* | *Depth* | *FACluster* | *%Dive* | *Season* | *tagFSA* | *tripDur* | *r^2^_m_* | *r^2^_c_* | *df* | *logLik* | *AICc* | *delta* | *weight* |
| --- | --- | --- | --- | --- | --- | --- | --- | --- | --- | --- | --- | --- | --- | --- |
| 41 | 7.61 | NA | NA | NA | + | NA | -0.15 | 0.17 | 0.2 | 5 | -54.62 | 120.67 | 0 | 0.21 |
| 9 | 6.61 | NA | NA | NA | + | NA | NA | 0.1 | 0.26 | 4 | -56.7 | 122.33 | 1.66 | 0.09 |
| 42 | 7.86 | 0 | NA | NA | + | NA | -0.17 | 0.18 | 0.18 | 6 | -54.39 | 122.83 | 2.16 | 0.07 |
| 45 | 7.68 | NA | NA | -0.01 | + | NA | -0.15 | 0.17 | 0.2 | 6 | -54.6 | 123.24 | 2.57 | 0.06 |
| 57 | 7.55 | NA | NA | NA | + | 0 | -0.15 | 0.17 | 0.2 | 6 | -54.61 | 123.26 | 2.59 | 0.06 |
| 33 | 7.79 | NA | NA | NA | NA | NA | -0.13 | 0.06 | 0.06 | 4 | -57.66 | 124.25 | 3.58 | 0.04 |
| 43 | 7.46 | NA | + | NA | + | NA | -0.15 | 0.2 | 0.21 | 7 | -53.74 | 124.28 | 3.61 | 0.04 |
| 13 | 6.8 | NA | NA | -0.01 | + | NA | NA | 0.1 | 0.26 | 5 | -56.58 | 124.59 | 3.92 | 0.03 |
| 25 | 6.46 | NA | NA | NA | + | 0.02 | NA | 0.1 | 0.26 | 5 | -56.59 | 124.6 | 3.93 | 0.03 |
| 44 | 7.89 | -0.01 | + | NA | + | NA | -0.18 | 0.24 | 0.24 | 8 | -52.56 | 124.8 | 4.13 | 0.03 |
| 1 | 6.94 | NA | NA | NA | NA | NA | NA | 0 | 0.02 | 3 | -59.14 | 124.82 | 4.15 | 0.03 |
| 10 | 6.59 | 0 | NA | NA | + | NA | NA | 0.1 | 0.26 | 5 | -56.7 | 124.82 | 4.15 | 0.03 |
| 36 | 8.01 | -0.01 | + | NA | NA | NA | -0.16 | 0.19 | 0.19 | 7 | -54.17 | 125.15 | 4.48 | 0.02 |
| 11 | 6.47 | NA | + | NA | + | NA | NA | 0.14 | 0.26 | 6 | -55.67 | 125.39 | 4.72 | 0.02 |
| 58 | 7.77 | -0.01 | NA | NA | + | 0.01 | -0.17 | 0.18 | 0.18 | 7 | -54.34 | 125.49 | 4.82 | 0.02 |
| 46 | 7.94 | 0 | NA | -0.01 | + | NA | -0.17 | 0.18 | 0.18 | 7 | -54.36 | 125.52 | 4.86 | 0.02 |
| 35 | 7.49 | NA | + | NA | NA | NA | -0.12 | 0.13 | 0.13 | 6 | -55.89 | 125.83 | 5.16 | 0.02 |
| 3 | 6.67 | NA | + | NA | NA | NA | NA | 0.08 | 0.11 | 5 | -57.23 | 125.9 | 5.23 | 0.02 |
| 61 | 7.62 | NA | NA | -0.01 | + | 0.01 | -0.15 | 0.18 | 0.2 | 7 | -54.58 | 125.95 | 5.29 | 0.02 |
| 34 | 8.04 | -0.01 | NA | NA | NA | NA | -0.15 | 0.07 | 0.07 | 5 | -57.41 | 126.25 | 5.58 | 0.01 |
| 37 | 7.96 | NA | NA | -0.01 | NA | NA | -0.12 | 0.07 | 0.07 | 5 | -57.53 | 126.48 | 5.81 | 0.01 |
| 5 | 7.22 | NA | NA | -0.02 | NA | NA | NA | 0.01 | 0.02 | 4 | -58.89 | 126.7 | 6.03 | 0.01 |
| 49 | 7.86 | NA | NA | NA | NA | -0.01 | -0.13 | 0.06 | 0.06 | 5 | -57.64 | 126.7 | 6.04 | 0.01 |
| 29 | 6.66 | NA | NA | -0.01 | + | 0.02 | NA | 0.11 | 0.26 | 6 | -56.45 | 126.96 | 6.29 | 0.01 |
| 4 | 6.83 | -0.01 | + | NA | NA | NA | NA | 0.11 | 0.12 | 6 | -56.5 | 127.05 | 6.38 | 0.01 |
| 47 | 7.56 | NA | + | -0.01 | + | NA | -0.15 | 0.21 | 0.21 | 8 | -53.69 | 127.06 | 6.4 | 0.01 |
| 59 | 7.53 | NA | + | NA | + | -0.01 | -0.15 | 0.21 | 0.21 | 8 | -53.72 | 127.13 | 6.46 | 0.01 |
| 17 | 6.92 | NA | NA | NA | NA | 0 | NA | 0 | 0.01 | 4 | -59.13 | 127.2 | 6.53 | 0.01 |
| 2 | 6.96 | 0 | NA | NA | NA | NA | NA | 0 | 0.02 | 4 | -59.13 | 127.2 | 6.53 | 0.01 |
| 14 | 6.79 | 0 | NA | -0.01 | + | NA | NA | 0.1 | 0.26 | 6 | -56.58 | 127.21 | 6.54 | 0.01 |
| 26 | 6.46 | 0 | NA | NA | + | 0.02 | NA | 0.1 | 0.26 | 6 | -56.59 | 127.22 | 6.55 | 0.01 |
| 12 | 6.6 | -0.01 | + | NA | + | NA | NA | 0.15 | 0.25 | 7 | -55.36 | 127.53 | 6.86 | 0.01 |
| 40 | 8.19 | -0.01 | + | -0.02 | NA | NA | -0.16 | 0.2 | 0.2 | 8 | -54 | 127.69 | 7.02 | 0.01 |
| 48 | 8.01 | -0.01 | + | -0.01 | + | NA | -0.18 | 0.25 | 0.25 | 9 | -52.49 | 127.71 | 7.04 | 0.01 |
| 60 | 7.88 | -0.01 | + | NA | + | 0 | -0.18 | 0.24 | 0.24 | 9 | -52.56 | 127.85 | 7.18 | 0.01 |
| 15 | 6.68 | NA | + | -0.01 | + | NA | NA | 0.14 | 0.26 | 7 | -55.53 | 127.86 | 7.19 | 0.01 |
| 52 | 8.09 | -0.01 | + | NA | NA | -0.01 | -0.16 | 0.19 | 0.19 | 8 | -54.13 | 127.94 | 7.28 | 0.01 |
| 7 | 6.95 | NA | + | -0.02 | NA | NA | NA | 0.09 | 0.11 | 6 | -56.98 | 128 | 7.34 | 0.01 |
| 27 | 6.42 | NA | + | NA | + | 0.01 | NA | 0.14 | 0.26 | 7 | -55.66 | 128.11 | 7.44 | 0.01 |
| 51 | 7.69 | NA | + | NA | NA | -0.02 | -0.13 | 0.14 | 0.14 | 7 | -55.71 | 128.21 | 7.54 | 0 |

Table S4. Summary of model parameters for all models included in model averaging for daily mass change of lactating northern fur seals in 1995 and 1996.

| *Model* | *(Intercept)* | *FACluster* | *InitMass* | *SeaFMRkg* | *Season* | *tagFSA* | *tripDur* | *r^2^* | *r^2^_adj_* | *df* | *logLik* | *AICc* | *delta* | *weight* |
| --- | --- | --- | --- | --- | --- | --- | --- | --- | --- | --- | --- | --- | --- | --- |
| 9 | 0.54 | NA | NA | NA | + | NA | NA | 0.12 | 0.1 | 3 | -21.88 | 50.31 | 0 | 0.16 |
| 41 | 0.82 | NA | NA | NA | + | NA | -0.04 | 0.14 | 0.11 | 4 | -21.1 | 51.12 | 0.81 | 0.1 |
| 11 | 1.01 | NA | -0.01 | NA | + | NA | NA | 0.14 | 0.1 | 4 | -21.23 | 51.39 | 1.08 | 0.09 |
| 25 | 0.39 | NA | NA | NA | + | 0.02 | NA | 0.14 | 0.1 | 4 | -21.26 | 51.45 | 1.14 | 0.09 |
| 27 | 0.9 | NA | -0.01 | NA | + | 0.02 | NA | 0.17 | 0.11 | 5 | -20.39 | 52.21 | 1.9 | 0.06 |
| 43 | 1.27 | NA | -0.01 | NA | + | NA | -0.04 | 0.17 | 0.11 | 5 | -20.46 | 52.34 | 2.03 | 0.06 |
| 13 | 0.68 | NA | NA | -0.02 | + | NA | NA | 0.12 | 0.08 | 4 | -21.83 | 52.6 | 2.29 | 0.05 |
| 57 | 0.65 | NA | NA | NA | + | 0.01 | -0.04 | 0.16 | 0.1 | 5 | -20.64 | 52.71 | 2.4 | 0.05 |
| 45 | 1.22 | NA | NA | -0.05 | + | NA | -0.05 | 0.15 | 0.1 | 5 | -20.81 | 53.06 | 2.75 | 0.04 |
| 15 | 1.32 | NA | -0.01 | -0.04 | + | NA | NA | 0.14 | 0.09 | 5 | -21.07 | 53.57 | 3.26 | 0.03 |
| 59 | 1.14 | NA | -0.01 | NA | + | 0.02 | -0.04 | 0.19 | 0.11 | 6 | -19.82 | 53.68 | 3.37 | 0.03 |
| 29 | 0.56 | NA | NA | -0.03 | + | 0.02 | NA | 0.14 | 0.08 | 5 | -21.18 | 53.79 | 3.48 | 0.03 |
| 1 | 0.71 | NA | NA | NA | NA | NA | NA | 0 | 0 | 2 | -24.83 | 53.92 | 3.61 | 0.03 |
| 47 | 1.91 | NA | -0.01 | -0.07 | + | NA | -0.05 | 0.18 | 0.11 | 6 | -19.94 | 53.93 | 3.62 | 0.03 |
| 31 | 1.28 | NA | -0.02 | -0.05 | + | 0.02 | NA | 0.18 | 0.1 | 6 | -20.14 | 54.33 | 4.02 | 0.02 |
| 61 | 1.07 | NA | NA | -0.06 | + | 0.01 | -0.05 | 0.17 | 0.09 | 6 | -20.34 | 54.72 | 4.41 | 0.02 |
| 10 | 0.53 | + | NA | NA | + | NA | NA | 0.12 | 0.06 | 5 | -21.73 | 54.89 | 4.58 | 0.02 |
| 3 | 1.15 | NA | -0.01 | NA | NA | NA | NA | 0.02 | 0 | 3 | -24.33 | 55.21 | 4.89 | 0.01 |
| 63 | 1.81 | NA | -0.02 | -0.08 | + | 0.02 | -0.05 | 0.21 | 0.11 | 7 | -19.22 | 55.23 | 4.92 | 0.01 |
| 33 | 0.9 | NA | NA | NA | NA | NA | -0.03 | 0.01 | -0.01 | 3 | -24.52 | 55.59 | 5.28 | 0.01 |
| 42 | 0.84 | + | NA | NA | + | NA | -0.05 | 0.15 | 0.08 | 6 | -20.8 | 55.64 | 5.33 | 0.01 |
| 17 | 0.63 | NA | NA | NA | NA | 0.01 | NA | 0.01 | -0.01 | 3 | -24.63 | 55.8 | 5.49 | 0.01 |
| 5 | 0.51 | NA | NA | 0.03 | NA | NA | NA | 0 | -0.02 | 3 | -24.75 | 56.04 | 5.73 | 0.01 |
| 12 | 0.99 | + | -0.01 | NA | + | NA | NA | 0.14 | 0.06 | 6 | -21.08 | 56.21 | 5.9 | 0.01 |
| 26 | 0.39 | + | NA | NA | + | 0.02 | NA | 0.14 | 0.06 | 6 | -21.2 | 56.45 | 6.14 | 0.01 |
| 19 | 1.09 | NA | -0.01 | NA | NA | 0.01 | NA | 0.03 | -0.01 | 4 | -24.04 | 57 | 6.69 | 0.01 |
| 35 | 1.32 | NA | -0.01 | NA | NA | NA | -0.03 | 0.03 | -0.01 | 4 | -24.04 | 57.02 | 6.71 | 0.01 |
| 44 | 1.26 | + | -0.01 | NA | + | NA | -0.05 | 0.18 | 0.08 | 7 | -20.21 | 57.21 | 6.9 | 0 |
| 14 | 0.7 | + | NA | -0.03 | + | NA | NA | 0.12 | 0.04 | 6 | -21.66 | 57.37 | 7.06 | 0 |
| 28 | 0.9 | + | -0.01 | NA | + | 0.02 | NA | 0.17 | 0.07 | 7 | -20.36 | 57.53 | 7.21 | 0 |

Table S5. Summary of model parameters for all models included in model averaging for absolute mass change of lactating northern fur seals in 1995 and 1996.

| *Model* | *(Intercept)* | *FACluster* | *InitMass* | *SeaFMRkg* | *Season* | *tagFA* | *tripDur* | *r^2^* | *r^2^_adj_* | *df* | *logLik* | *AICc* | *delta* | *weight* |
| --- | --- | --- | --- | --- | --- | --- | --- | --- | --- | --- | --- | --- | --- | --- |
| 41 | -0.34 | NA | NA | NA | + | NA | 0.6 | 0.26 | 0.22 | 4 | -108.17 | 225.26 | 0 | 0.26 |
| 57 | -1.68 | NA | NA | NA | + | 0.12 | 0.64 | 0.28 | 0.23 | 5 | -107.32 | 226.08 | 0.81 | 0.18 |
| 43 | 0.83 | NA | -0.03 | NA | + | NA | 0.6 | 0.26 | 0.21 | 5 | -108.05 | 227.53 | 2.27 | 0.08 |
| 45 | 0.75 | NA | NA | -0.14 | + | NA | 0.57 | 0.26 | 0.21 | 5 | -108.11 | 227.66 | 2.39 | 0.08 |
| 59 | -0.14 | NA | -0.04 | NA | + | 0.13 | 0.64 | 0.29 | 0.22 | 6 | -107.1 | 228.25 | 2.99 | 0.06 |
| 33 | 0.13 | NA | NA | NA | NA | NA | 0.68 | 0.16 | 0.15 | 3 | -110.95 | 228.45 | 3.19 | 0.05 |
| 61 | -0.5 | NA | NA | -0.16 | + | 0.12 | 0.61 | 0.28 | 0.22 | 6 | -107.26 | 228.57 | 3.31 | 0.05 |
| 42 | -0.46 | + | NA | NA | + | NA | 0.59 | 0.26 | 0.19 | 6 | -107.92 | 229.89 | 4.63 | 0.03 |
| 47 | 2.53 | NA | -0.04 | -0.19 | + | NA | 0.57 | 0.26 | 0.19 | 6 | -107.96 | 229.97 | 4.71 | 0.03 |
| 49 | -0.8 | NA | NA | NA | NA | 0.08 | 0.71 | 0.18 | 0.14 | 4 | -110.57 | 230.08 | 4.82 | 0.02 |
| 9 | 3.42 | NA | NA | NA | + | NA | NA | 0.13 | 0.11 | 3 | -111.83 | 230.2 | 4.94 | 0.02 |
| 37 | -1.6 | NA | NA | 0.22 | NA | NA | 0.71 | 0.17 | 0.13 | 4 | -110.82 | 230.58 | 5.32 | 0.02 |
| 35 | 1.13 | NA | -0.03 | NA | NA | NA | 0.68 | 0.17 | 0.13 | 4 | -110.88 | 230.69 | 5.42 | 0.02 |
| 63 | 1.83 | NA | -0.05 | -0.23 | + | 0.13 | 0.61 | 0.29 | 0.21 | 7 | -106.97 | 230.74 | 5.47 | 0.02 |
| 58 | -1.62 | + | NA | NA | + | 0.11 | 0.63 | 0.28 | 0.2 | 7 | -107.26 | 231.32 | 6.06 | 0.01 |
| 13 | 6.69 | NA | NA | -0.49 | + | NA | NA | 0.15 | 0.12 | 4 | -111.23 | 231.39 | 6.13 | 0.01 |
| 34 | -0.38 | + | NA | NA | NA | NA | 0.68 | 0.19 | 0.13 | 5 | -110.23 | 231.89 | 6.63 | 0.01 |
| 25 | 2.71 | NA | NA | NA | + | 0.08 | NA | 0.14 | 0.11 | 4 | -111.52 | 231.97 | 6.7 | 0.01 |
| 53 | -2.62 | NA | NA | 0.23 | NA | 0.08 | 0.74 | 0.18 | 0.13 | 5 | -110.43 | 232.29 | 7.03 | 0.01 |
| 51 | 0.46 | NA | -0.04 | NA | NA | 0.09 | 0.72 | 0.18 | 0.13 | 5 | -110.45 | 232.32 | 7.06 | 0.01 |
| 44 | 0.71 | + | -0.03 | NA | + | NA | 0.6 | 0.27 | 0.18 | 7 | -107.8 | 232.4 | 7.14 | 0.01 |
| 46 | 1.05 | + | NA | -0.2 | + | NA | 0.56 | 0.27 | 0.18 | 7 | -107.82 | 232.44 | 7.17 | 0.01 |
| 11 | 4.43 | NA | -0.03 | NA | + | NA | NA | 0.14 | 0.1 | 4 | -111.76 | 232.45 | 7.19 | 0.01 |

Table S6. Summary of model parameters for all models included in model averaging for water influx of lactating northern fur seals in 1995 and 1996, including marginal (*r^2^_m_*) and conditional (*r^2^_c_*) estimates.

| *Model* | *(Intercept)* | *FACluster* | *InitMass* | *SeaFMRkg* | *Season* | *tagFA* | *tripDur* | *r^2^m* | *r^2^c* | *df* | *logLik* | *AICc* | *delta* | *weight* |
| --- | --- | --- | --- | --- | --- | --- | --- | --- | --- | --- | --- | --- | --- | --- |
| 4 | 270.8 | + | -2.3 | NA | NA | NA | NA | 0.43 | 0.73 | 6 | -216.5 | 447.04 | 0 | 0.27 |
| 36 | 250.41 | + | -2.3 | NA | NA | NA | 3.05 | 0.46 | 0.73 | 7 | -215.35 | 447.49 | 0.45 | 0.21 |
| 8 | 309.93 | + | -2.47 | -4.8 | NA | NA | NA | 0.47 | 0.67 | 7 | -216.03 | 448.87 | 1.82 | 0.11 |
| 40 | 283.24 | + | -2.46 | -3.84 | NA | NA | 2.9 | 0.49 | 0.69 | 8 | -215.01 | 449.71 | 2.66 | 0.07 |
| 12 | 271.1 | + | -2.3 | NA | + | NA | NA | 0.43 | 0.72 | 7 | -216.49 | 449.78 | 2.73 | 0.07 |
| 20 | 270.97 | + | -2.3 | NA | NA | -0.02 | NA | 0.43 | 0.73 | 7 | -216.5 | 449.79 | 2.75 | 0.07 |
| 44 | 249.83 | + | -2.31 | NA | + | NA | 3.32 | 0.46 | 0.73 | 8 | -215.2 | 450.1 | 3.05 | 0.06 |
| 52 | 248 | + | -2.33 | NA | NA | 0.23 | 3.14 | 0.46 | 0.73 | 8 | -215.31 | 450.32 | 3.28 | 0.05 |
| 16 | 312.18 | + | -2.49 | -5.15 | + | NA | NA | 0.47 | 0.67 | 8 | -216.01 | 451.71 | 4.67 | 0.03 |
| 24 | 310.47 | + | -2.47 | -4.81 | NA | -0.06 | NA | 0.47 | 0.67 | 8 | -216.03 | 451.75 | 4.71 | 0.03 |
| 28 | 271.56 | + | -2.3 | NA | + | -0.06 | NA | 0.43 | 0.72 | 8 | -216.49 | 452.67 | 5.62 | 0.02 |
| 56 | 280.74 | + | -2.48 | -3.78 | NA | 0.2 | 2.99 | 0.49 | 0.69 | 9 | -214.98 | 452.7 | 5.66 | 0.02 |
| 48 | 279.33 | + | -2.44 | -3.4 | + | NA | 3.04 | 0.49 | 0.69 | 9 | -214.98 | 452.7 | 5.66 | 0.02 |
